# Understudied factors in drug‐coated balloon design and evaluation: A biophysical perspective

**DOI:** 10.1002/btm2.10370

**Published:** 2022-07-07

**Authors:** Tarek Shazly, William M. Torres, Eric A. Secemsky, Vipul C. Chitalia, Farouc A. Jaffer, Vijaya B. Kolachalama

**Affiliations:** ^1^ College of Engineering & Computing University of South Carolina Columbia South Carolina USA; ^2^ Exponent Inc. Philadelphia Pennsylvania USA; ^3^ Smith Center for Outcomes Research in Cardiology Beth Israel Deaconess Medical Center Boston Massachusetts USA; ^4^ Department of Medicine, Boston University School of Medicine Boston Veterans Affairs Healthcare System Boston Massachusetts USA; ^5^ Cardiovascular Research Center and Cardiology Division Massachusetts General Hospital Boston Massachusetts USA; ^6^ Department of Medicine, Boston University School of Medicine; Department of Computer Science and Faculty of Computing & Data Sciences Boston University Boston Massachusetts USA

**Keywords:** arterial disease, drug‐coated balloon, stenosis, vascular drug delivery

## Abstract

Drug‐coated balloon (DCB) percutaneous interventional therapy allows for durable reopening of the narrowed lumen via physical tissue expansion and local anti‐restenosis drug delivery, providing an alternative to traditional uncoated balloons or a permanent indwelling implant such as a conventional metallic drug‐eluting stent. While DCB‐based treatment of peripheral arterial disease (PAD) has been incorporated into clinical guidelines, DCB use has been recently curtailed due to reports that showed evidence of increased mortality risk in patients treated with paclitaxel (PTX)‐coated balloons. Given the United States Food and Drug Administration's 2019 consequent warning regarding PTX‐eluting DCBs and the subsequent marked reduction in clinical DCB use, there is now a critical need to better understand the compositional and mechanical factors underlying DCB efficacy and safety. Most work to date on DCB refinement has focused on designing both the enabling balloon catheter and alternate coatings composed of various drugs and excipients, followed by device evaluation in preclinical and clinical studies. We contend that improvement in DCB performance will require a better understanding of the biophysical factors operative during and following balloon deployment, and moreover that the elaboration and demonstrated control of these factors are needed to address current concerns with DCB use. This article provides a perspective on the biophysical interactions that govern DCB performance and offers new design strategies for the development of next‐generation DCB devices.

## 1 INTRODUCTION

The prevalence and significance of peripheral artery disease (PAD) continue to increase in our aging population and remains a major cause of critical limb ischemia and amputations.^1^, ^2^, ^3^ However, current endovascular interventions for PAD, such as percutaneous transluminal angioplasty (PTA) alone or in conjunction with endovascular stents, are limited by high restenosis rates ranging from 39% to 60% at 1 year.^4^, ^5^ Moreover, long lesions within the infrainguinal vasculature and device fracture rates limit the efficacy of stent‐based technologies.^6^, ^7^ Drug‐coated balloon (DCB) therapy, which is an angioplasty balloon‐based treatment that delivers an antiproliferative/anti‐restenosis payload to the artery wall, has become the effective standard‐of‐care treatment for PAD,^8^, ^9^, ^10^ and an emerging choice for in‐stent restenosis and de novo lesions within the coronary vasculature, due to their clinical effectiveness and avoidance of a permanent metal implant such as a stent, and superiority to conventional uncoated balloon angioplasty. However, in late 2018, Katsanos et al. published a provocative meta‐analysis of randomized controlled trials of paclitaxel (PTX)‐coated peripheral DCBs,^11^ demonstrating that DCB‐treated patients experienced an increased late mortality rate. Although plagued by missing data, the investigators found that in 12 studies reporting survival at 2 years, there was an absolute risk difference (DCB harm) of 3.5%, which approached 7% by 4–5 years in three studies. Following the publication of this unexpected result, in 2019 the United States Food and Drug Administration (FDA) subsequently advised interventionalists to utilize DCBs in only the highest‐risk patients followed by close post‐procedural follow‐up.^12^ This decision has markedly reduced clinical DCB use,^13^ potentially leading to higher restenosis rates following endovascular interventions that now rely on conventional balloons or bare metal stents.

Notably, a causal mechanism linking PTX‐based DCBs and mortality has yet to be determined, which is critical in understanding the validity of the statistical signal. Attempts to establish a dose–response relationship have been unsuccessful,^14^ and specific causes of death between those exposed to PTX‐coated devices versus uncoated devices have not revealed a causal association.^15^ Both the failure to determine a mechanistic relationship with DCBs and mortality, coupled with the dramatic FDA response to the Katsanos' study, underscores our limited understanding of how DCB devices work and the conditions that can lead to untoward scenarios. While emerging data suggests the potential for PTX‐based DCBs to be safe clinically,^16^, ^17^, ^18^ and to serve as a plaque stabilizing therapy,^19^ the presently halted use of DCBs serves as an impetus to obtain a deeper understanding of the mechanisms governing DCB function and define the role of biophysical factors in balloon transfer of free drug and/or coating to the arterial wall, and subsequent drug transport, retention, and distribution.

Several factors spanning the design (preprocedural), deployment (intra‐procedural), and follow‐up (post‐procedural) phases need to be understood to fully understand and evaluate DCB efficacy and safety. With a goal of maximizing the degree of anti‐restenosis agent (e.g., PTX) transfer and retention into the target lesion, many studies have focused on optimizing the design of the balloon catheter, including its geometry and elastic mechanical properties, as well as alternate excipient‐drug combinations for coating formation.^20^ A few studies have also explored antiproliferative compounds belonging to the “limus” family as drugs of choice for DCB therapy.^21^, ^22^, ^23^, ^24^ Additionally, other studies have focused on lesion preparation prior to DCB deployment,^25^, ^26^, ^27^ with the goals of modifying calcified plaque, facilitating balloon expansion, and promoting maximal DCB‐surface contact with the arterial wall. The design of device constituents and strategies for lesion preparation are key factors associated with the pre‐procedural phase. The post‐procedural phase has been examined in both experimental and clinical settings, in which identified endpoints/factors include the degree of restored lumen patency, off‐target drug effects, the efficiency of drug delivery, dissolution, convection, diffusion and drug binding,^21^ barriers to absorption, and interaction between the drug, delivery vehicle, and overall drug pharmacokinetics within the arterial wall.^28^

Conversely to these well‐studied pre‐ and post‐procedural phases, we submit that the intra‐procedural phase, where the device meets the artery, has been understudied. Moreover, evidence suggests that the role of underlying biophysical events operative during this phase can be generalized across candidate DCBs and will prove to be deterministic of drug transfer and overall device efficacy. Below we identify the general problems limiting current DCBs, highlight key factors and findings germane to the pre‐ and post‐procedural phases, present a detailed breakdown of the dynamic interactions during DCB deployment by viewing it primarily as a sequence of biophysical events, and propose future directions to improve DCB efficacy.

## 2 CURRENT DCB DESIGNS AND ORIGINS OF RISK

### 2.1 Approved DCBs

There are several experimental, animal, and human studies focused on DCB design, development, and evaluation.^29^ DCBs have been considered for deployment in the coronary circulation (to treat both in‐stent restenosis and de novo lesions; mainly outside the United States),^30^, ^31^, ^32^, ^33^ in the peripheral circulation (for both femoropopliteal [within the United States] and below‐the‐knee indications),^34^, ^35^, ^36^, ^37^, ^38^, ^39^ and to a lesser degree in arteriovenous fistula and grafts (AVF/AVG).^40^, ^41^, ^42^, ^43^ While it is not practical to cover all potential clinical applications in this article, we highlight some of the well‐known DCBs that have been approved by the FDA for primary treatment of femoropopliteal disease.^44^

- IN.PACT™ Admiral™: The IN.PACT Admiral DCB is indicated for de novo, restenotic, or in‐stent restenotic lesions up to 360 mm in length with reference vessel diameters of 4–7 mm in the superficial femoral or popliteal arteries. The balloon surface of IN.PACT has a nominal paclitaxel dose density of 3.5 μg/mm^2^ and uses urea as the excipient. In the IN.PACT superficial femoral artery (SFA) trial,^37^ two‐year outcomes demonstrated a durable and superior treatment effect of DCB versus plain‐old transluminal angioplasty (PTA) with significantly higher primary patency, lower clinically‐driven target lesion revascularization, and similar functional status improvement with fewer repeat interventions. The 5‐year outcomes of this trial were also similar, demonstrating IN.PACT Admiral DCB's superior performance over PTA.^45^
- Lutonix®: Similar to IN.PACT, this DCB catheter is also indicated for native superficial femoral or popliteal vessel disease containing lesions up to 300 mm in length with reference vessel diameters of 4–7 mm. The surface of this catheter uses a combination of polysorbate and sorbitol along with paclitaxel dose density of 2 μg/mm^2^. The Lutonix Paclitaxel‐Coated Balloon for the Prevention of Femoropopliteal Restenosis (LEVANT I) trial showed that composite two‐year adverse events were 39% for the DCB group compared to 46% for the PTA group, suggesting that Lutonix DCBs had safety comparable to that of the control group.^38^
- Stellarex™: This DCB is coated with polyethylene glycol 8000 along with paclitaxel dose density of 2 μg/mm^2^. This device was approved for native lesions up to 180 mm in length with reference vessel diameters of 4–6 mm. In the ILLUMENATE Pivotal Study,^36^ the rate of clinically driven target lesion revascularization was significantly lower in the DCB cohort (7.9% versus 16.8%), demonstrating superior safety and effectiveness of the Stellarex DCB in comparison with PTA.
- Ranger™: The device was approved for lesions with up to 180 mm in length within reference vessel diameters of 4–7 mm. As above, this device also contains paclitaxel with a nominal dose density of 2 μg/mm^2^, and acetyl tributyl citrate, which is a plasticizer as its excipient. In the RANGER SFA trial,^39^ the DCB group had a greater primary patency rate at 12 months compared to the control group, and this result was associated with low revascularization rate and good clinical outcomes.

### 2.2 Drug and coating transfer inefficiencies reduce DCB efficacy

**FIGURE 1:**
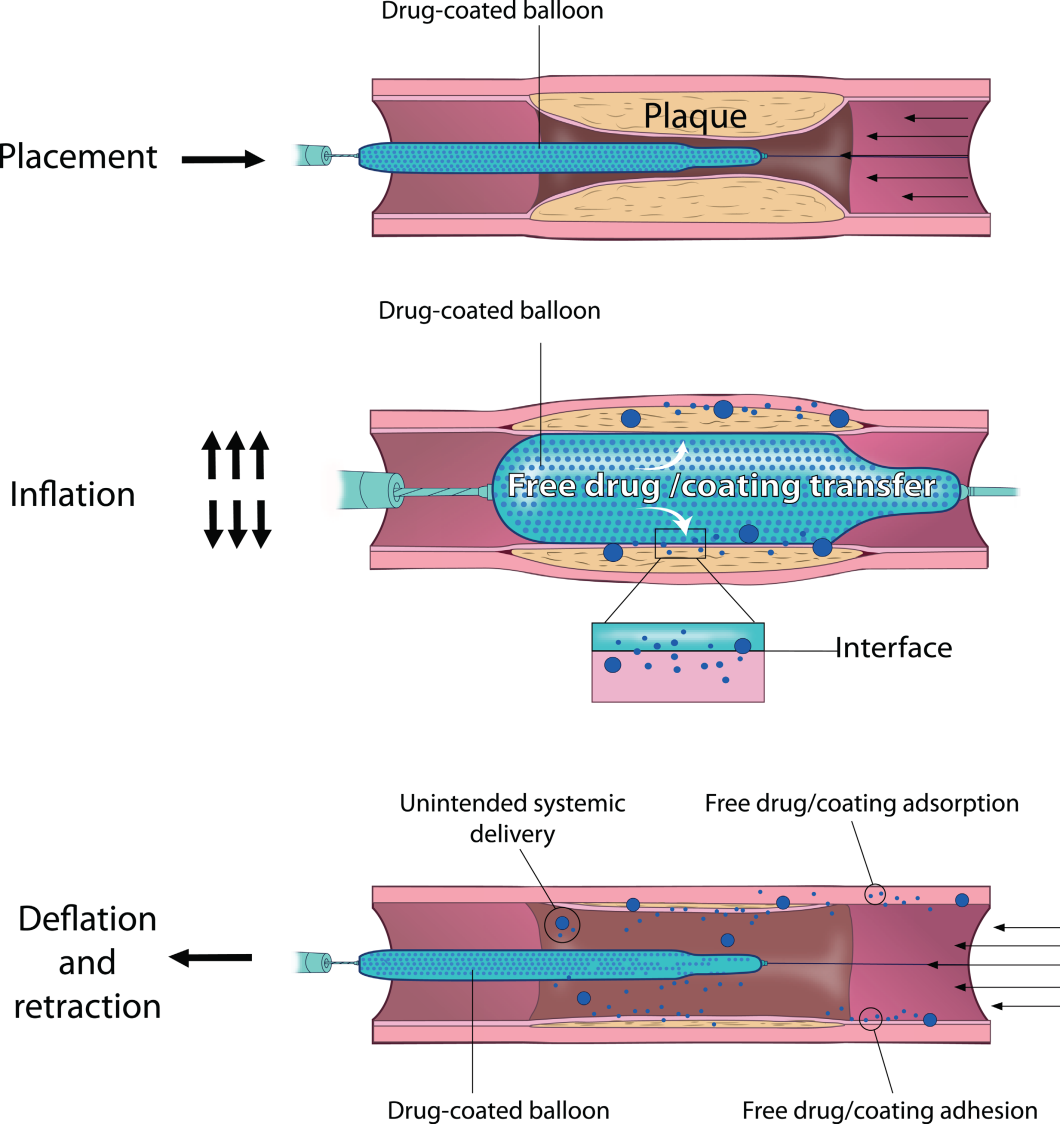
Stages of DCB deployment. The DCB procedure includes balloon placement at the target site (top), balloon inflation (middle), and balloon deflation/retraction (bottom). During placement, the balloon is guided to and positioned at the site of plaque accumulation. During inflation, the plaque is compressed, and coating constituents (coating/drug) are transferred from the balloon surface to the arterial wall. During deflation/retraction, some transferred constituents adhere to/are adsorbed by the arterial wall, while some is lost to the circulation.

As noted above, all FDA‐approved DCBs contain PTX as their drug component, with various excipients that serve as drug carriers. During the procedure, the balloon catheter advances from the arterial insertion site to the target artery application site. During this step, a reasonable amount of coating/drug can potentially be lost to the circulation, representing the first mode of efficiency reduction.^46^ After the balloon reaches the site and during the balloon inflation, the coating/drug undergoes a net diffusive transport from the catheter to the vessel wall due to the concentration gradient established across the coating‐vessel interface (Figure 1). Despite significant excipient/coating variability among proposed DCBs, all devices exhibit inefficient total drug transfer to the tissue during the procedural window (approximately <10% of total drug delivered on‐target).^47^ Moreover, a few pre‐clinical studies have shown that only a small portion of the coating (~8%) is transferred during balloon inflation,^48^ and almost 90% of the delivered PTX releases from the arterial wall into the systemic circulation within 48 hours, increasing the potential for systemic toxicity.^47^ To compensate for inefficient drug transfer and subsequent wash‐off, coatings are designed with high initial drug concentrations that ensure therapeutic dosing is nevertheless achieved. Indeed, a multitude of previous studies have shown that with this approach, drug concentrations in local tissue are such that tissue binding capacity is reached within seconds after balloon inflation.^49^ Due to PTX's well‐known pharmacological properties, namely its binding/transport kinetics and high binding specificity,^50^, ^51^, ^52^ the antiproliferative effects subsequently persist for prolonged periods. However, we postulate that the compensatory design strategy of high initial coating drug content comes at a cost and identify it as a primary limitation of current DCBs. Specifically, high balloon concentrations and inefficient delivery together result in higher amount of drug release into the circulation and drug accumulation in distal/off‐target sites, and thus elevate the risk of adverse systemic events.

At the coating level (as opposed to drug level), balloon inflation and appropriate sizing of the balloon with the artery causes the coating to interact both chemically (i.e., adhesive bond formation) and mechanically (i.e., coating/tissue deformation, coating penetration into the tissue) with the intraluminal (or endothelial) surface. These interactions partially determine coating fate upon balloon deflation, wherein coating‐tissue adhesion exceeding innate coating adhesion to the balloon surface promotes coating transfer to the tissue. Here of relevance are the solvents and additives used to develop PTX‐based coatings, which directly influence coating stability and drug transfer rates.^53^ Transferred coating, if adsorbed and retained at the target site, can then serve as a reservoir for continued drug delivery and may extend the treatment window in comparison to free drug delivery. However, initially transferred coating that later is dislodged by the convective forces of pulsatile blood flow may lead to drug/coating embolization, a process which represents a second major risk factor associated with DCB use. In analogy to drug transfer efficiency, increasing the efficiency of coating transfer to the vessel wall would enable DCB designs with less coating content and thus lower embolic potential.

Taken together, the *transfer inefficiencies* of DCB constituents (drug and coating) to the arterial wall are likely culprits in limiting DCB efficacy. Thus, while there are numerous directions for DCB refinement, including strategies for patient‐ and lesion‐specific dosing, alternative drugs for DCB applications, and excipient selection, enhancing drug and coating transfer efficiency can be viewed as an independent performance objective that can be readily quantified and used as a basis for iterative device design.

## 3 BIOPHYSICAL FACTORS IN DCB DEPLOYMENT

### 3.1 DCB deployment procedure as a sequence of biophysical events

From introduction into the circulation to retraction from the patient, DCBs undergo a series of biophysical events that cumulatively influence the efficiency of drug/coating transfer to the arterial wall. Firstly, the catheter guidance to and positioning within the targeted vasculature relies on adequate device stiffness and steerability. Although not directly determinate of transfer efficiency, precision placement and control of device positioning is a prerequisite for successful intervention. Next, upon unsheathing, the coating is exposed to local flow‐induced shear stress that promotes constituent wash‐off and mass loss to the circulation. Once the balloon is positioned and unsheathed, the dynamic inflation process is initiated, and a coating‐arterial wall interface is formed. Subsequent balloon inflation (beyond that required for initial contact up to that required to reach the balloon operating diameter) transmits a radial force through this interface and results in a bi‐axial deformation of the arterial wall, characterized by a radial compression and circumferential tension that reestablishes the arterial lumen. The coating also undergoes circumferential tension during balloon inflation, and depending on its microstructure and mechanical properties, may also experience a radial compression upon contact with the arterial wall. In comparison to physiological arterial loading, complete balloon inflation is achieved under extremely high inflation pressures.^54^ As a result, in the fully inflated configuration when the balloon outer diameter equals the artery inner diameter, this dimension represents a kinematic constraint on the arterial deformation. Even when appropriately sized balloons are used to proportionally match the vessel size, the arterial wall exhibits significant viscoelastic behavior in non‐physiological loading domains, and this imposed kinematic constraint may induce a mechanical creep response that modulates the interface throughout the inflation period. Taken together, these complex mechanical events will dictate the transient coating‐arterial wall interface operative during DCB inflation.

**FIGURE 2:**
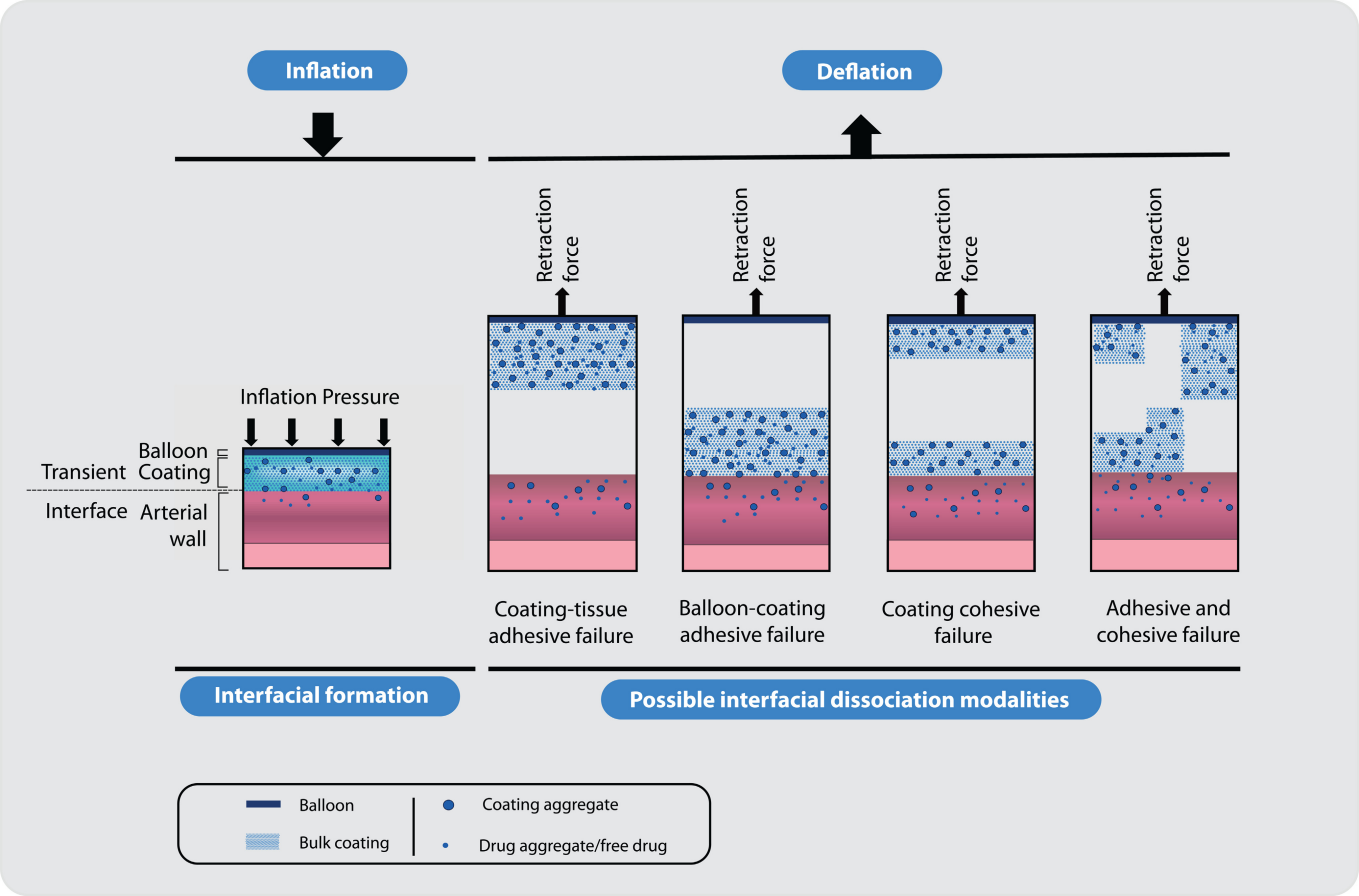
Interfacial mechanisms during DCB angioplasty. While the interfacial bond is formed between the coating and the artery during balloon inflation, there can be a few possible ways by which the bond failure can occur during balloon deflation.

Via this dynamic interface that the functionally central diffusive transport of drug from the coating to the arterial wall occurs, the deformation of both the coating and arterial wall will dictate key geometric factors in drug/coating transfer efficiencies, namely coating‐artery contact area and coating penetration depth into the arterial wall. Upon balloon deflation/device retraction, the adhesive forces developed between the coating and arterial wall are placed in conflict with the cohesive forces in the coating itself and the adhesive force between the coating and the balloon, wherein the relative magnitude of these forces will determine if coating is transferred and ultimately retained at the treatment site (Figure 2). In the following sections, we will consider each of these biophysical events in detail and focus on their potential role in drug/coating transfer efficiency with DCB deployment.

### 3.2 Interfacial formation and dissociation

**FIGURE 3:**
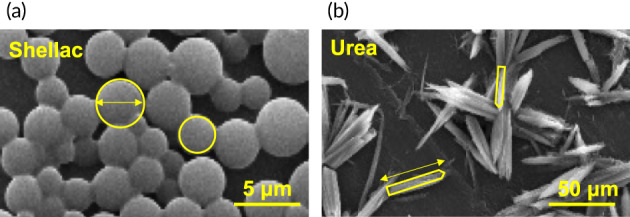
Intrinsic shape of the balloon coatings. Image derived from scanning electron microscopy show spherical structures for shellac (a) and conical structures for urea (b). The coatings were developed at room temperature by mixing paclitaxel with the respective materials in 1:1 ratio, using a micro‐pipetting method. Nylon‐12 was used as the balloon material. Processed from data published in Chang et al.^20^

At the microstructural level, current coatings have significant geometrical variance which in turn impacts contact mechanics and interfacial formation with arterial tissue during DCB inflation.^20^ For example, experimental urea‐based coatings have a conical, needle‐like microstructure, while shellac‐based coatings are largely composed of spherical elements (Figure 3). Moreover, these microstructural geometries present a range of characteristic length scales that would further impact local coating‐tissue interactions. In the formation of the tissue‐coating interface, it is likely that the needle‐like urea microstructure, particularly for the subset of aggregate coating domains oriented perpendicularly to the balloon surface, will promote penetration into the tissue due to a small contact area over which the inflation force is transmitted. Conversely, a spherical microstructure will have a comparatively high contact area but have less tendency to penetrate the arterial wall. While the impact of these microstructural differences on drug transfer and contact mechanics has been explained for a subset of experimental coatings,^20^ it is not immediately clear which of these phenomena will ultimately lead to enhanced therapeutic gains, as both drug delivery at a greater depth within the arterial wall (due to coating penetration) or through a greater contact area (due to a lack of coating penetration) can presumably enhance drug transfer efficiency.

Along with drug transfer, interfacial formation also potentiates coating transfer to the vessel wall. An ideal DCB deployment would entail interfacial continuity during the inflation phase, but also adhesion/adsorption of the coating upon interfacial dissociation. With the transfer of drug laden coating, these domains can serve as vehicles for protracted drug delivery and drug residence time. To meet the objective of coating transfer, the strength of adhesion of the coating to the vessel wall during balloon deflation/ interfacial dissociation must dominate other concurrent interactions, i.e., those between the coating and the balloon (coating‐balloon adhesion) as well as within the coating itself (coating cohesion). Indeed, preliminary studies have shown that simple surface modification of the balloon catheter can facilitate coating transfer to the vessel wall by weakening balloon‐coating adhesion and suggest that such strategies have potential for broad application across candidate DCB designs.^55^

## 4 BIOPHYSICS‐BASED DESIGN CONSIDERATIONS AND STRATEGIES

### 4.1 Designing a viable option for lumen reopening

Regardless of the mode of lesion preparation, DCB catheters must be capable of achieving full inflation within the narrowed lumen via controlled device pressurization, ensuring that a continuous/nearly continuous interface is formed with the arterial wall. This aspect of procedural success is critical, as a DCB procedure cannot be considered superior to plain‐old angioplasty without completion of this primary task. Clearly, this implies that balloons must be designed such that they allow for safe, pressure‐based inflation under various conditions of lumen geometry/plaque burden.

Pressure‐based inflation of balloon catheters remained the mainstay and essentially has not changed since the first set of devices were deployed using angioplasty decades ago. Even today, interventionalists routinely use this technique by following the same process of using a high‐pressure inflator to open the narrowed artery. While it is understandable that such high pressures must be developed for arterial reopening in the presence of stiff plaque, current pressure‐based inflators are simply single‐step devices with analog gauges, and they do not provide enough fidelity to gradually increase or decrease the pressure during inflation and deflation, respectively. Previous studies have shown that higher inflation pressures and more compliant balloon materials cause markedly large surface‐contact areas and contact stresses in the context of stents.^56^ However, there is insufficient evidence to indicate that controlled inflation or deflation can improve outcomes. We argue that studies that can evaluate better control on the pressure gauge need to be conducted to identify balloon inflation tools as an additional lever during the procedure. Similarly, the interplay among balloon compliance, coating transfer, and drug delivery needs to be further studied. Focus needs to be also directed on evaluating and improving the angioplasty balloon surface properties, as recent studies have shown an increase in drug delivery efficiency with chemically‐modified balloon surfaces.^55^ Such efficiency can potentially reduce the amount of requisite initial drug loading, thereby lowering the risk of excessive wash‐out of the drug after the procedure, minimizing the risk of embolization and reducing systemic toxicity of DCB therapy.

### 4.2 Optimizing the surface of the balloon catheter

**FIGURE 4:**
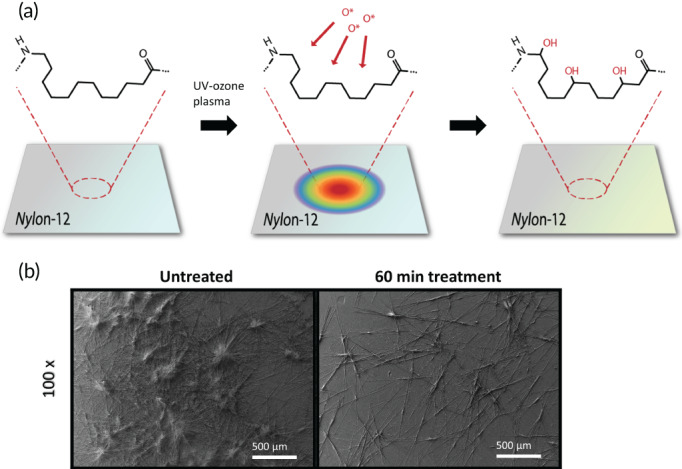
Surface modification of balloon catheters. (a) Oxygen‐based functional groups were added to the polymer molecules present on the Nylon‐12 films by exposing them to atomic oxygen in the chamber. (b) Scanning electron microscopy images of untreated and ultraviolet‐ozone plasma treated balloon surfaces. Obtained from Azar et al.^55^

Owing to the short procedural time, drug doses on the balloon catheters are an order‐of‐magnitude higher than typically used in drug‐eluting stents.^57^ The question then arises as to whether such high doses can create untoward events such as local and systemic toxicity. To potentially minimize these issues, one could focus on modifying the design of the balloon catheters with lower doses of drug and/or identify alternative modalities to facilitate efficient drug delivery. A recent study focused on modifying the surface of the balloon catheter using ultraviolet‐ozone plasma (UVO) treatment (Figure 4), which increased the hydrophilicity of the balloon surface and altered the microstructure of the coating material, leading to improved transfer of drug‐laden coating to the artery.^55^ Although biocompatibility of such engineered balloon surfaces remains to be evaluated, design strategies to alter the biophysical properties of the canonical balloon material can be further explored.

### 4.3 Providing a durable post‐procedural anti‐restenotic effect

Once the balloon is deflated, both transferred and residual coating domains are exposed to the hemodynamic environment. The post‐deployment coating, in either domain, can remain on the respective surface only if the operative cohesive/adhesive forces resist dissolution and wash‐off. Mechanical failure in this regard may cause a downstream reaction, include off‐site drug effects and embolic development. Therefore, designing a coating that can withstand the pulsatile forces of blood, even when in the fractured or particulate form, is critical to long‐term safety of the DCB procedure. This implies that extensive bench‐top studies need to be performed that can simulate a post‐procedural environment where different adhered coatings are tested to withstand the flow over time. Coatings that can withstand such testing need to be isolated for further studies.

### 4.4 Lesion preparation

Vessel preparation has gained importance as a crucial component of endovascular procedures such as DCB angioplasty. Typically, a semi‐ or a non‐compliant balloon with a balloon‐artery ratio of 1:1 is used for lesion preparation, prior to DCB application. However, in some cases, smaller balloon sizes are used along with drugs such as vasodilators for lesion preparation. Also, in lesions that are anticipated to be stiff, high‐pressure induced non‐compliant or cutting and scoring balloons are used. Similarly, adjunctive procedures such as atherectomy (laser, rotational, orbital) or intravascular lithotripsy can be used to ablate or modify the lesion to facilitate optimal balloon expansion and subsequent drug delivery. Recent studies have shown that adequate lesion pretreatment enhances the drug penetration into the vessel wall, which promotes and increases the anti‐restenotic properties.^25^ We postulate that such improved efficiency can be attributed to the changes in the biophysical footprint of the prepared vessel in contrast to the lesion with unexpanded plaque. While studies have indicated that vessel preparation minimizes the risk of dissections, maximizes the luminal gain, and prepares the vessel bed for local drug delivery,^26^, ^27^ it is important to also quantify the biophysical significance of lesion preparation, which can inform better DCB design.

### 4.5 Bench‐top studies in DCB design and evaluation

**FIGURE 5:**
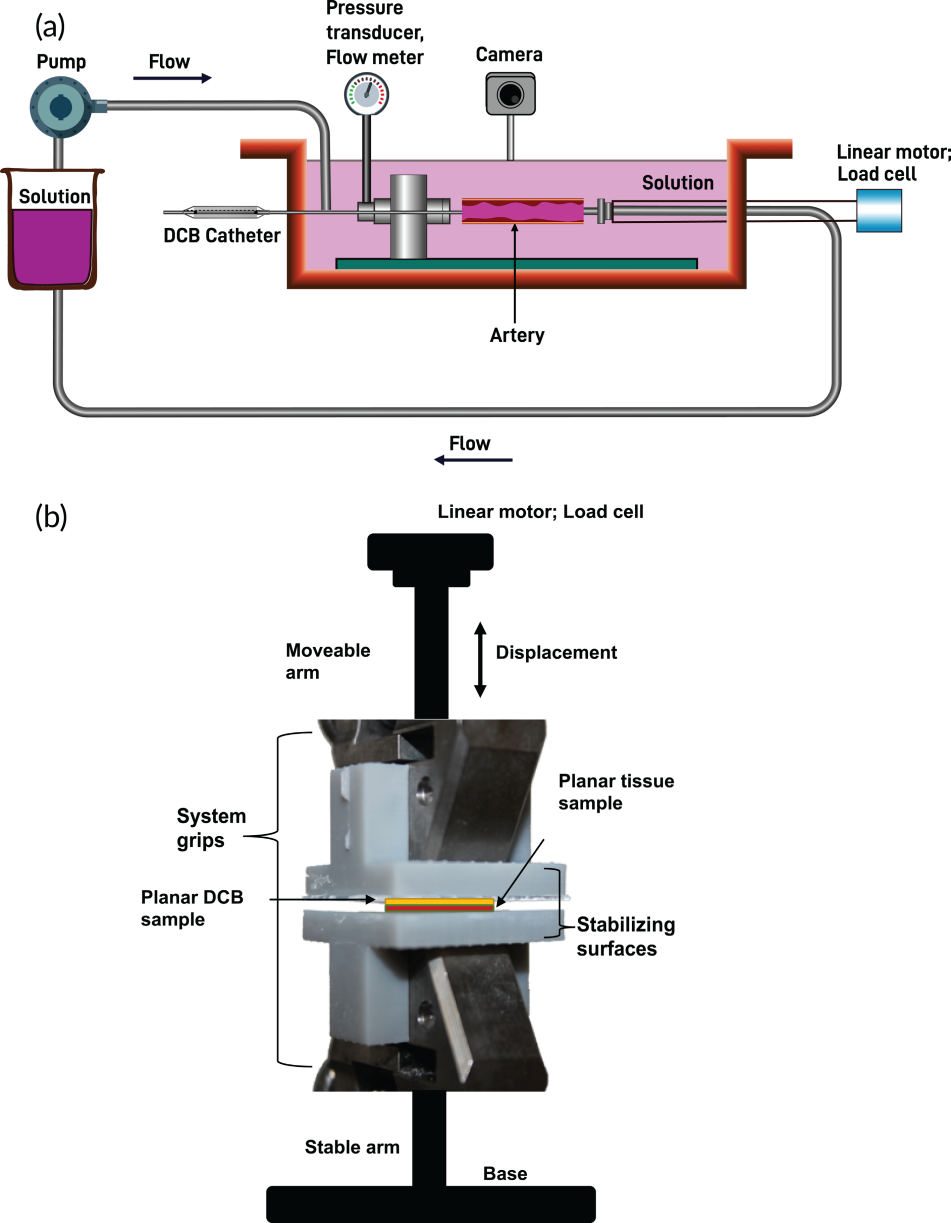
Bench‐top studies in DCB design and evaluation. (a) Candidate DCBs could be deployed and evaluated in an ex vivo flow system, with essential system components including a pump/pressure transducer to facilitate controlled flow, a linear motor/load cell to impart/monitor axial loads, a camera to measure vessel deformation, and an access port for catheter placement in a vascular test segment. (b) Candidate coatings could also be evaluated in a uniaxial test set‐up, in which planar tissue and coatings samples are placed in controlled contact under mechanical loading scenarios that simulate the biophysical interactions operative during DCB deployment.

Accepting the notion that intra‐procedural transfer efficiencies of DCB constituents (drug and coating) to the arterial wall are deterministic factors governing current DCB efficacy, it is rational to incorporate bench‐top studies that quantify constituent transfer within the early stages of DCB development and design. Metrics of constituent transfer should be assessed in the context of an experimental set‐up which approximates the biophysical factors operative during DCB deployment, including both contact forces developed with the arterial wall and blood flow‐derived shear forces. Procedural approximation could be achieved via DCB inflation within an ex vivo flow system with arterial or venous flow rates as indicated, in which vessel/device geometries, the mode of tissue‐device interaction (balloon inflation), and local hemodynamics can be faithfully represented (Figure 5a). Alternatively, drug/coating transfer could be evaluated in a comparatively high‐throughput uniaxial test set‐up,^20^ in which the one‐dimensional nature/planar geometry of tissue‐device interaction is significantly different from actual device deployment conditions but contact forces can nevertheless be controlled (Figure 5b).

Irrespective of the mode by which DCB deployment is approximated in bench‐top studies, subsequent analyses that quantify constituent transfer to and retention within the arterial tissue will provide key response variables to guide iterative device design. In terms of coating composition and transfer, scanning electron microscopy (SEM) images of the coating surface before and after controlled interaction with arterial tissue can be processed to quantify coating dissociation from the balloon,^20^, ^55^, ^58^, ^59^ while analogous wet SEM imaging of the tissue surface can elucidate coating retention within the arterial wall. To assess drug transfer efficiency, acutely delivered drug levels can be measured via high‐performance liquid chromatography (HPLC) of arterial tissue samples following simulated DCB deployment and compared to the initial drug content of the coating. Clearly, bench‐top methods to simulate DCB deployment and associated measures of coating/drug transfer efficiency will quantitatively differ from analogous in vivo measures,^46^ but trend identification with novel coatings and iterative coating designs (i.e., variable excipient/drug mass ratio) represent a powerful tool for early‐stage device development.^60^

### 4.6 Computational modeling in DCB design and evaluation

**FIGURE 6:**
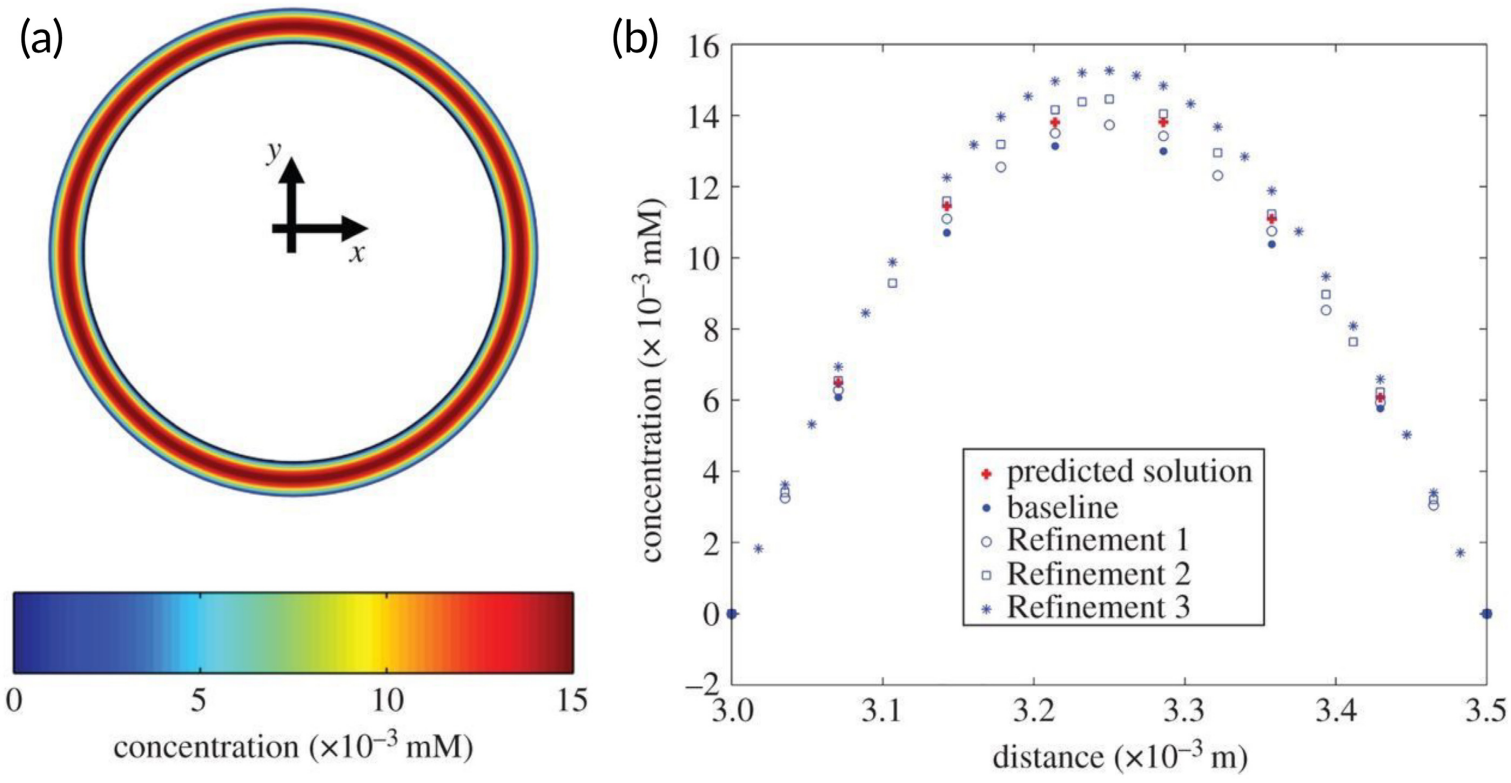
Physics‐based modeling of drug‐coated balloon delivery. (a) True solution for the bound drug was computed on the mesh setting with the highest density. (b) Arterial vessel concentration for bound drug for solutions based on four mesh configurations and a supervised learning model‐predicted solution were plotted as a function of cross‐sectional depth. Reproduced from Kolandaivelu et al.^69^

With advancements in both numerical methods and hardware capabilities over the last several decades, the value proposition of computational modeling and simulation tools for the analysis of implantable medical devices has been on the rise.^61^ This technology provides researchers with the ability to create controlled experiments to parse out the interplay between all the design and response variables for a given device—all with an increase in efficiency and decrease in cost compared to traditional benchtop studies. To date, computational modeling and simulation has been widely used to evaluate drug‐eluting stents to facilitate optimization of their performance.^62^ These studies have served to elucidate on the importance of considerations for the impact of luminal flow characteristics on drug‐elution,^63^, ^64^ how the optimal rate of elution is dependent on drug composition,^65^ how the strut position and/or design has an effect on the spatial distribution of deposited drug in areas not directly in contact with the stent, and many other components of their design. Conversely, the adoption of these methods for the study of drug transport from drug‐coated balloons has been relatively slow with only a small number of studies published on the subject. Several studies have investigated the role of the heterogeneous atherosclerotic lesion on the eventual efficacy of the therapy.^66^, ^67^, ^68^ In a 2013 study, computational modeling was performed to explore the pharmacokinetics of zotarolimus as a therapeutic agent instead of paclitaxel, the drug used in almost every other study.^21^ Additionally, Kolandaivelu et al. presented a supervised machine learning framework, using drug coated balloons as an exemplary scenario (Figure 6), to process data generated from coarse meshes to predict results derived from highly‐refined meshes – thereby drastically increasing the efficiency in the computational modeling workflow.^69^ However, it must be noted that all the studies to‐date, except one,^70^ are inherently limited by the fact that the geometric domain is simplified down to a one‐ or two‐dimensional representation. While it has been shown that lower‐dimensional models can adequately predict the numerical results that would be derived from three‐dimensional models when the device‐vessel interface is spatially uniform, the assumption begins to break down as heterogeneity in both the vessel tissue and balloon surface are incorporated into the computational model.^71^

Given the complex interplay between the balloon design, flow, and drug parameters, there is an opportunity for computational modeling and simulation to be deployed in sophisticated optimization strategies for targeting improved device performance. As an example, we know that sufficient inflation pressure is required to both break through stiff atherosclerotic lesions and ensure proper contact area with the balloon surface. However, this high inflation pressure comes along with the risk of acute trauma to the blood vessel or even severe dissection.^72^ Future studies focused on evaluating the relationship between inflation pressure and the local stress within the arterial wall would reveal the proper operating conditions for inflation pressure along with lesion preparation to ensure acute safety and efficacy. Furthermore, given the short residence time in the lumen as compared to drug‐eluting stents, drug‐eluting/coated balloons usually rely on high doses of drug coated along the exterior surface of the balloon. As a result, care must be taken to design coatings with sufficient adherence to the balloon to prevent wash‐off of the coating resulting in systemic toxicity and/or embolism. To address this concern, computational fluid dynamics studies could be deployed to provide insight into the shear stresses that the surface is exposed to in the period between unsheathing of the balloon and the moment it meets the vessel wall to obtain performance benchmarks for the strength of the coating adhesion. Finally, it is well understood that the efficacy of drug deposition is highly dependent on the contact pressure between the balloon and the surface of the lesion. Lee et al. presented a novel balloon design with a linear micropattern along the surface of the balloon and demonstrated that this design resulted in higher drug deposition in vivo as compared to traditional designs with a smooth cylindrical surface.^73^ Taken together with our understanding that the microstructural elements of the drug coating can be modulated based on its composition,^20^ it stands to reason that there is an optimal solution which appropriately strikes the balance between micro‐scale surface roughness and contact area.

## 5 CONCLUSION

Endovascular devices have undergone various iterations over the past decades, with each generation attempting to address the limitations of the previous one. The fundamental goal of durably reopening the narrowed vessel lumen with procedural, biological, and clinical success remains unchanged. The recent shortcomings of DCB technology should be viewed as opportunities to rethink the design strategies by focusing on mechanistic aspects that can improve the design of next generation balloon catheters. The physical properties of the balloon, coating, and arterial wall, along with the manner of inflation and deflation, will cumulatively determine the interfacial interactions operative throughout the DCB procedure. Biophysical mechanisms of drug transfer, delivery, and retention are thus far underexplored in the DCB design cycle, and we submit that a focus on interfacial mechanics in conjunction with experimental strategies incorporating in vivo imaging and computational modeling can help us quantify the drivers of DCB performance.

## AUTHOR CONTRIBUTIONS

**Tarek Shazly:** Conceptualization (equal); data curation (equal); investigation (equal); methodology (equal); writing – original draft (equal); writing – review and editing (equal). **William Torres:** Writing – review and editing (supporting). **Eric A. Secemsky:** Writing – review and editing (supporting). **Vipul C. Chitalia:** Writing – review and editing (supporting). **Farouc A. Jaffer:** Writing – review and editing (supporting). **Vijaya B. Kolachalama:** Conceptualization (equal); data curation (equal); funding acquisition (lead); investigation (equal); methodology (equal); project administration (lead); supervision (lead); writing – original draft (equal); writing – review and editing (equal).

### PEER REVIEW

The peer review history for this article is available at https://publons.com/publon/10.1002/btm2.10370.

## Data Availability

Data sharing is not applicable to this article as no datasets were generated or analyzed during the current study.
